# Integrating deep learning with ECG, heart rate variability and demographic data for improved detection of atrial fibrillation

**DOI:** 10.1136/openhrt-2025-003185

**Published:** 2025-03-31

**Authors:** Araz Rawshani, Aidin Rawshani, Gustav Smith, Jan Boren, Deepak L Bhatt, Mats Börjesson, Johan Engdahl, Peter Kelly, Antros Louca, Truls Ramunddal, Erik Andersson, Elmir Omerovic, Zacharias Mandalenakis, Vibha Gupta

**Affiliations:** 1 Departement of Clinical & Molecular Medicine, Institute of Medicine, Gothenburg, Sweden; 2 University of Gothenburg Institute of Medicine, Goteborg, Sweden; 3 Department of Cardiology, Sahlgrenska University Hospital, Goteborg, Sweden; 4 Icahn School of Medicine at Mount Sinai, New York, NY, USA; 5 Department of Molecular and Clinical Medicine, Gothenburg University, Gothenburg, Sweden; 6 Department of Clinical and Molecular Medicine, University of Gothenburg Institute of Medicine, Goteborg, Sweden

**Keywords:** Atrial Fibrillation, Atrial Flutter, Electrocardiography

## Abstract

**Background:**

Atrial fibrillation (AF) is a common but often undiagnosed condition, increasing the risk of stroke and heart failure. Early detection is crucial, yet traditional methods struggle with AF’s transient nature. This study investigates how augmenting ECG data with heart rate variability (HRV) and demographic data (age and sex) can improve AF detection.

**Methods:**

We analysed 35 634 12-lead ECG recordings from three public databases (China Physiological Signal Challenge-Extra, PTB-XL and Georgia), each with physician-validated AF labels. A range of convolutional neural network models, including AlexNet, VGG-16, ResNet and transformers, were tested for AF prediction, enriched with HRV and demographic data to explore the effectiveness of the multimodal approach. Each data modality (ECG, HRV and demographic) was assessed for its contribution to model performance using fivefold cross-validation. Performance improvements were evaluated across key metrics, and saliency maps were generated to provide further insights into model behaviour and identify critical features in AF detection.

**Results:**

Integrating HRV and demographic data with ECG substantially improved performance. AlexNet and VGG-16 outperformed more complex models, achieving AUROC of 0.9617 (95% CI 0.95 to 0.97) and 0.9668 (95% CI 0.96 to 0.97), respectively. Adding HRV data showed the most significant improvement in sensitivity, with AlexNet increasing from 0.9117 to 0.9225 and VGG-16 from 0.9216 to 0.9225. Combining both HRV and demographic data led to further improvements, with AlexNet achieving a sensitivity of 0.9225 (up from 0.9192 with HRV) and VGG-16 reaching 0.9113 (up from 0.9097 with HRV). The combination of HRV and demographic data resulted in the highest gains in sensitivity and area under the receiver operating characteristic curve. Saliency maps confirmed the models identified key AF features, such as the absence of the P-wave, validating the multimodal approach.

**Conclusions:**

AlexNet and VGG-16 excelled in AF detection, with HRV data improving sensitivity, and demographic data providing additional benefits. These results highlight the potential of multimodal approaches, pending further clinical validation.

**WHAT IS ALREADY KNOWN ON THIS TOPIC**

- Atrial fibrillation (AF) is often undiagnosed, contributing to increased risks of stroke and heart failure. Current detection methods struggle with AF’s transient nature, and there has been limited exploration of augmenting ECG data with heart rate variability (HRV) and demographic factors to improve detection accuracy.

**WHAT THIS STUDY ADDS**

- This study demonstrates that combining ECG data with HRV and demographic information significantly improves AF detection accuracy. By leveraging multimodal data, including HRV and demographic factors, the models show enhanced sensitivity and robustness, revealing critical features of AF.

**HOW THIS STUDY MIGHT AFFECT RESEARCH, PRACTICE OR POLICY**

- Integrating interpretable features into AF detection models can enhance clinical decision-making by providing clear, actionable insights into the key aspects of AF. This multimodal approach not only improves detection accuracy but also promotes transparency and interpretability in machine learning models. These advancements could inform the development of evidence-based guidelines for early AF detection and treatment strategies, ultimately improving patient care.

## Introduction

Atrial fibrillation (AF) presents significant health challenges by increasing the risk of stroke, heart failure and overall morbidity and mortality.^1^ Early detection of AF is pivotal in initiating timely interventions.^2^ AF detection remains challenging, primarily due to its often asymptomatic nature, transient and intermittent occurrence, and difficulties acquiring and evaluating long ECG recordings. Different studies^3^,^5^ estimate that 8%–48% of individuals with AF are asymptomatic, with prevalence varying based on patient population, age, comorbidities and study methodology. Large registry-based studies report lower prevalence (eg, 8.1%), while cardiology cohort studies estimate rates as high as 43%–48%. Epidemiological data further suggest that up to one-third of individuals with AF may not experience symptoms. Given the swift and broad introduction of consumer devices capable of detecting AF, it is pivotal to continue improving machine learning (ML) methods for this purpose.^6 7^

Theoretically, the clinical approach to diagnosing AF is based primarily on an irregular ventricular rhythm with an absence of P waves. Traditional ML methods^8^,^11^ for AF detection focus on specific features like P wave presence and RR interval (time between two consecutive R-wave peaks in the ECG signal) regularity, involving manual or semiautomated feature extraction followed by rule-based or statistical analysis. However, these traditional methods have significant limitations. They depend on accurately identifying these features and can be affected by noise, missing complex patterns in the data.^12^ Recent studies have shown that neural networks can analyse these features, improving on traditional methods. Hybrid models like U-Net and ResNet, as well as DenseNet, have been used to extract key ECG features, enhancing AF classification.^13^,^15^ Techniques like SHAP with Random Forests have also improved interpretability, while combining P wave analysis with RR interval variability helps reduce false positives.^16 17^ These advancements highlight the potential of deep learning (DL) for automating AF detection and improving clinical reliability.

DL models, unlike traditional methods, use the entire ECG signal, automatically identifying important features, handling noise more effectively and detecting intricate patterns. This makes them valuable tools for clinicians, facilitating more accurate diagnoses of AF. DL techniques, particularly convolutional neural networks (CNNs) and recurrent neural networks (RNNs), have demonstrated remarkable efficiency in analysing ECG data for AF detection on single-lead and multilead ECGs^18^,^21^ during normal sinus rhythm. Despite the widespread use of ECGs in clinical practice, much of the existing research on AF detection has primarily focused on single-lead ECG data.^22^,^24^ However, 12-lead ECGs provide a more comprehensive view of the heart’s electrical activity, offering richer information that can enhance AF diagnosis. Although studies leveraging the potential of 12-lead ECGs for AF detection are limited,^25^,^27^ their integration with advanced neural network architectures highlights the promise of DL models in delivering robust and accurate diagnoses.

Previous studies have largely focused on standalone DL models or the independent analysis of heart rate variability (HRV) features. In contrast, our research adopts a more holistic approach by explicitly incorporating HRV parameters into the modelling framework. While HRV features are inherently present in raw ECG data, we explicitly calculate and integrate these parameters during training to assess their additive predictive value. Using 12-lead ECG data, we evaluated several state-of-the-art DL architectures in both baseline and multimodal configurations, combining HRV parameters, demographic data (age and sex) and raw ECG signals. This multimodal approach is designed to enhance model performance and reliability in detecting AF. To validate the effectiveness of HRV and demographic features, we extended our methodology by employing extreme gradient boosting (XGBoost)^28^ as a downstream classifier. This approach allowed us to assess whether combining these features with the deep representations extracted by CNNs could improve AF detection. XGBoost, with its strength in handling structured data, enabled us to disentangle the individual contributions of HRV parameters and demographic factors in enhancing predictive performance.

## Methods

### Datasets

There are publicly available datasets^29^ corresponding to 12-lead ECGs, containing annotations for various classes, including AF. Instead of datasets exclusively dedicated to AF and sinus rhythm, these datasets encompass multiple classes, among which AF is included. In our research, we leveraged these datasets; sourced from various sites to ensure wide variability and treating AF and atrial flutter (AFL) as positive classes, while other rhythms were designated as negative classes. Although specific datasets like the MIT-BIH Atrial Fibrillation Database focus on single-lead ECG data for AF detection and are directly available. In this study, we used publicly available recordings, totalling 35 634 recordings of 12-lead ECGs from 3 databases. All recordings were sampled at a frequency of 500 Hz. The databases include diagnoses for AF and AFL but do not distinguish between symptomatic or asymptomatic AF. Therefore, our analysis focused on detecting the presence of AF and AFL, treating them as a composite endpoint, with AF being the predominant condition in the positive class.

The PTB-XL database contains 21 837 ECG recordings, of which 1570 had a diagnosis of AF or AFL. ECG records in PTB-XL were annotated by up to two cardiologists with up to 71 predefined statements conforming to the SCP-ECG standard. All recordings in PTB-XL were 10 s long.^30^ The China Physiological Signal Challenge Extra (CPSC Extra) dataset consists of 12-lead ECGs collected from 11 hospitals. All recordings ranged from 6 to 60 s in length. The dataset contains physician-labelled diagnoses for AF and AFL. Among 3453 recordings, 207 had AF or AFL.^31^ Finally, the Georgia 12-Lead-ECG database included 10 344 recordings, of which 741 had AF or AFL.^29^

For consistency, we focused only on the 10 s recordings from all three databases. All recordings were standardised to a common length of 10 s (5000 samples at a 500 Hz sampling rate), and signals exceeding 5000 samples were truncated. Data from these sources were integrated to ensure diversity in the training data, facilitating a robust evaluation of the model’s performance across a variety of cardiac conditions. Online supplemental table 1 provides a summary of the total number of recordings, the number of AF/non-AF cases, and the number of 10 s ECG segments used for training, validation and testing.

### Preprocessing and feature extraction

The raw 12-lead ECG signals consist of 12 time series, but only 8 leads provide unique and valuable data. Therefore, leads I, II and V1–V6 were retained for further analysis, while the remaining leads (III, aVF, aVR and aVL) were excluded, as they are derived from other leads and should, therefore, be captured by the neural networks. This reduction helped simplify the input while retaining crucial information for model training.

We then calculated the heart rate for each recording, applying wavelet denoising to reduce noise and improve the heart rate calculation. Since some leads may not provide reliable heart rate estimates due to noise or low amplitude,^32^ we computed the heart rate for each lead individually, allowing us to determine the heart rate for nearly all patients, except for 20 cases. The lead with the median heart rate was selected to calculate HRV parameters. The HRV parameters extracted included heart rate, interbeat interval (time between successive heartbeats), HRV-SDNN (SD of NN intervals), HRV-SD of successive differences between NN intervals, HRV-root mean square of successive differences between NN intervals, PNN20 (percentage of successive NN intervals differing by more than 20 ms), PNN50 (percentage of successive NN intervals differing by more than 50 ms) and HR MAD (heart rate mean absolute deviation). These HRV parameters were then integrated into the multimodal models to assess their additional contribution beyond the features extracted by the neural networks. Detailed filtration procedures and equations for HRV feature calculation are provided in the online supplemental materials under ‘Details Related to HRV Feature Calculation.’

### Modelling approach

We employed a comprehensive modelling approach that involved multiple techniques to enhance predictive performance. The first step involved using CNN models, which were trained solely on raw ECG signals. We implemented CNN models using various architectures, including Le-Net,^33^ AlexNet,^34^ VGG-16,^35^ ResNet50,^36^ ResNet,^37^ Inception,^38^ FCN,^39^ Encoder, Separable CNN, GRU^40^ and Transformers.^41^ Transformers, initially designed for natural language processing, have recently been deployed for ECG classification. Their self-attention mechanism enables them to process longer ECG sequences and capture complex temporal relationships that other architectures may overlook.^18^

**Figure 1:**
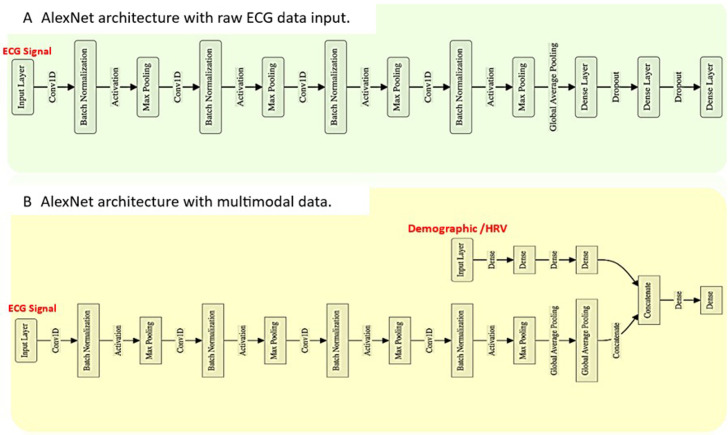
Schematic representation of the multimodal approach. (**A**) AlexNet architecture with raw ECG data input. (**B**) AlexNet architecture with multimodal data, incorporating ECG signals, demographic information (age, sex) and heart rate variability (HRV) features.

To further improve model performance, we integrated demographic and HRV data into the CNN models, creating multimodal architecture. Visual representation of the multimodal architecture is outlined in figure 1, using AlexNet as the example. This approach used two parallel input streams: one stream processed the raw ECG signals, while the other processed demographic data (such as age and sex) and HRV features. These two streams were combined through dense layers, enabling the model to handle both signal-based and non-signal-based data simultaneously. The goal was to leverage this additional context to enhance prediction accuracy.

In addition to the CNN models, we employed XGBoost as a downstream classifier to assess the contribution of HRV and demographic features. Features extracted from the final dense layers of the CNN models were concatenated with demographic and HRV data to form a comprehensive feature set, which was then input into the XGBoost model. This methodology allowed us to evaluate the added value of HRV and demographic features beyond the ECG signals alone.

### Training protocol

The dataset was divided into training (80%) and testing (20%) sets. The training set was further split into training and validation subsets using an 80/20 ratio. A fivefold cross-validation strategy was employed to ensure robust model evaluation and prevent overfitting. For the training of CNN models, the learning rate was dynamically adjusted using TensorFlow’s ReduceLROnPlateau^42^ function. This mechanism monitored validation loss during training and reduced the learning rate when progress plateaued, helping to optimise convergence.

To address the class imbalance between AF and non-AF classes, experiments were conducted with both class-weighted and non-class-weighted approaches. The class-weighted strategy assigned higher weights to underrepresented classes, mitigating imbalance issues. The non-class-weighted approach simulated real-world scenarios where imbalances are prevalent.

For the XGBoost modelling, hyperparameter optimisation was performed using the Optuna^43^ framework, which systematically fine-tuned parameters to maximise model performance. Early stopping was also implemented to prevent overfitting by halting training when validation performance ceased to improve.

### Performance metrics and model interpretability

Model performance was assessed using key metrics, including sensitivity, F-score, specificity, positive predictive value (PPV), negative predictive value (NPV) and the area under the receiver operating characteristic curve (AUC-ROC). The AUC-ROC provided a threshold-independent evaluation of the model’s ability to distinguish between classes across varying decision thresholds. 95% CIs were calculated for all primary performance metrics, ensuring the robustness of statistical interpretations. The mathematical equations for performance metrics such as accuracy, precision, recall, F1-score and AUC-ROC, along with their detailed explanations, have been included in the online supplemental materials under the section ‘Performance Metrics and Model Interpretability.’

For interpretability, saliency maps were employed to highlight the regions of the ECG signals that had the most influence on the model’s predictions. These visualisations offered insights into the decision-making process, improving transparency and facilitating potential clinical adoption.

## Results

All experiments were conducted using both class-weight and non-class-weight approaches to address the highly imbalanced dataset, where only 4.4% of samples belonged to the positive class. Training loss progression for AlexNet and VGG-16, across various data modalities, is shown in online supplemental figure 1. As observed, the loss decreased sharply after the initial epoch. For all scenarios, the training loss plateaued after approximately 6 epochs, with no signs of overfitting or underfitting. The code related to data handling, preprocessing and model development has been made publicly available on GitHub. The repository can be accessed here: https://github.com/Vibha190685/ECG-AI-AFib.

### Performance of individual CNN models: with ECG signal only

**Figure 2:**
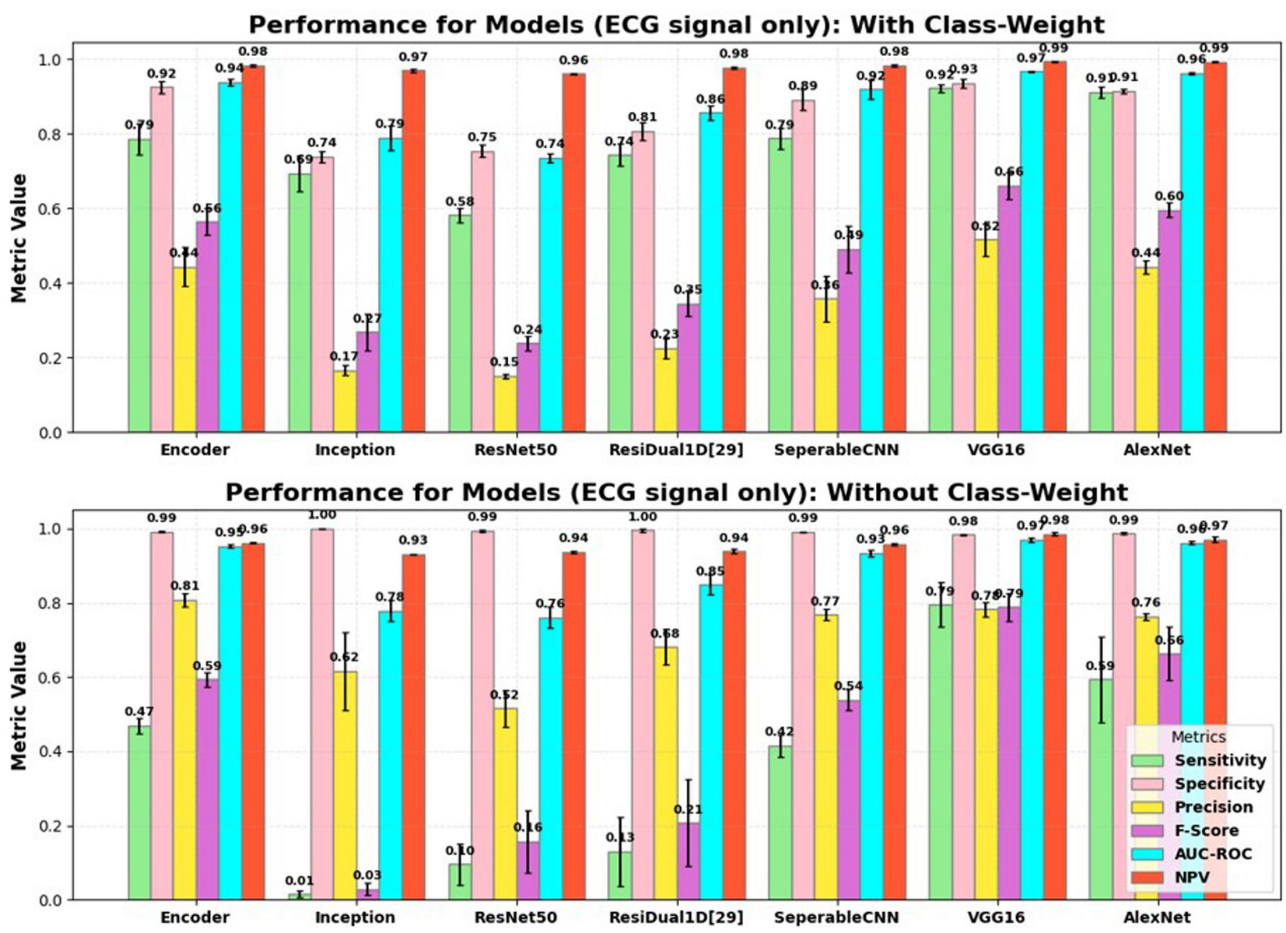
Performance comparison of deep learning models on ECG signals: (**A**) with class weight and (**B**) without class weight. AUC-ROC, area under the receiver operating characteristic curve; NPV, negative predictive value.

Figure 2 presents the performance of various CNN models on ECG data, both with and without class weights. The top-performing models were AlexNet and VGG-16. With class weighting, AlexNet achieved a sensitivity of 91.17%, specificity of 91.41%, precision of 44.26%, NPV of 99.28% and an AUC-ROC of 0.9617. VGG-16 achieved a sensitivity of 92.17%, specificity of 93.47%, precision of 51.60%, NPV of 99.38% and an AUC-ROC of 0.9669. Separable CNN also showed decent performance, with a sensitivity of 78.72%, specificity of 89.11% and an AUC-ROC of 0.9184. However, it trailed behind AlexNet and VGG-16 in terms of precision, sensitivity and F-score. Transformers exhibited the lowest performance across all metrics, achieving a sensitivity of 78.73%, an F-score of 0.4266 and an AUC-ROC of 0.9047.

When class weights were removed, both AlexNet and VGG-16 showed improvements in specificity and AUC-ROC but saw a significant drop in sensitivity. AlexNet’s sensitivity decreased to 59.34%, specificity increased to 98.63% and its AUC-ROC decreased slightly to 0.9612. VGG-16’s sensitivity dropped to 79.46%, specificity increased to 98.36% and AUC-ROC rose to 0.9704. Separable CNN’s sensitivity dropped to 41.53%, with specificity increasing to 99.06% and AUC-ROC decreasing to 0.9335. These results highlight the trade-off between sensitivity and other performance metrics. For clinical applications, particularly in AF detection, high sensitivity is crucial to minimise missed AF detections, while adjustments to specificity and AUC-ROC are also important for achieving a balanced model.

### Multimodal CNN models: impact of integrating HRV and demographic data to ECG

**Figure 3:**
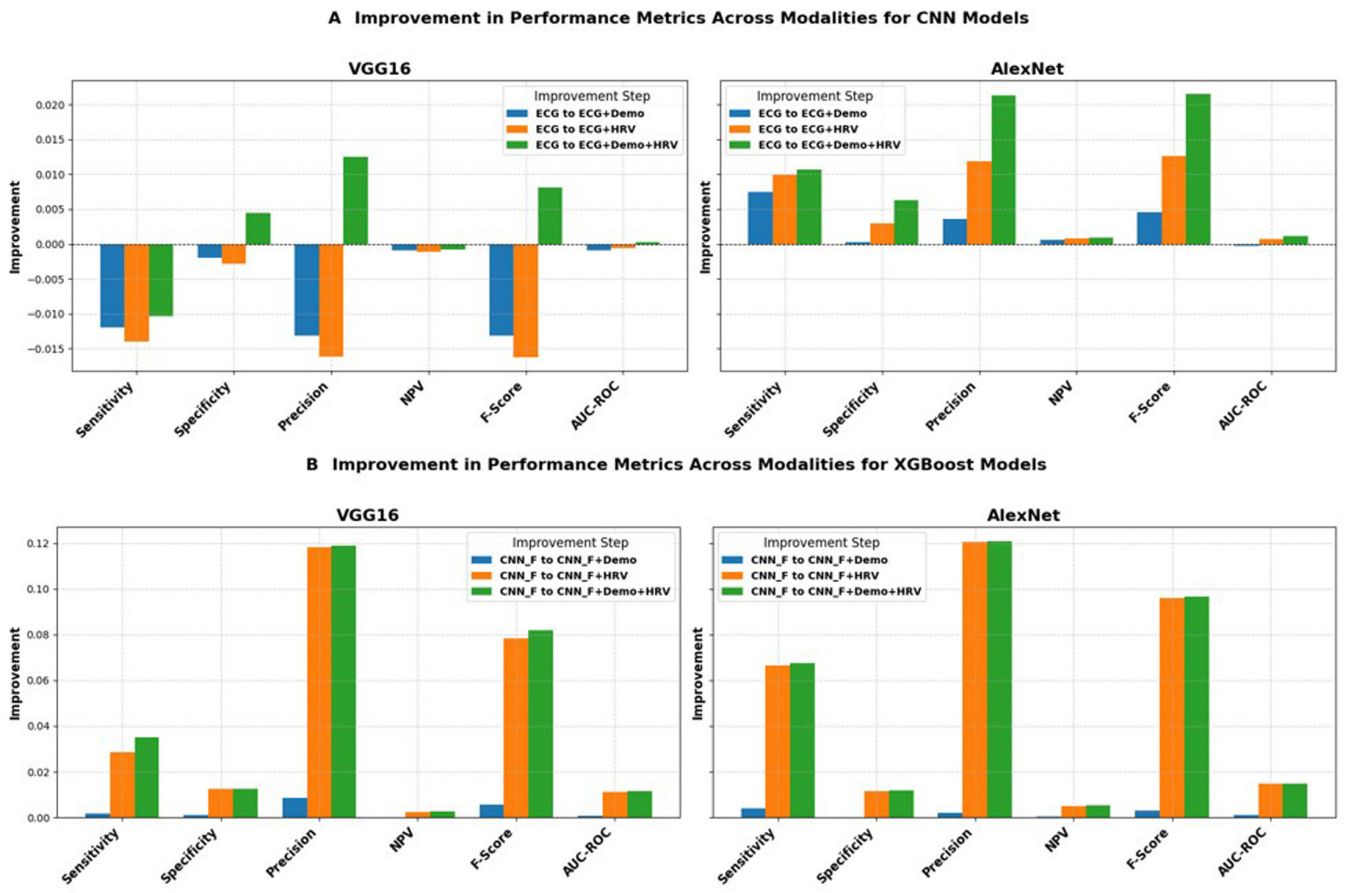
Impact of integrating multimodal data (ECG, demographic and HRV) on model performance across key metrics. (**A**) Trends in performance metrics (sensitivity, specificity, precision, NPV, F-Score, AUC-ROC) for CNN models, comparing ECG-only input to augmented datasets (ECG+Demo, ECG+HRV, ECG+Demo+HRV). (**B**) Performance analysis for XGBoost models using CNN-extracted features, highlighting improvements with added demographic (CNN_F+Demo), HRV (CNN_F+HRV) and combined data (CNN_F+Demo+HRV). AUC-ROC, area under the receiver operating characteristic curve; CNN, convolutional neural network; HRV, heart rate variability; NPV, negative predictive value.

**Figure 4:**
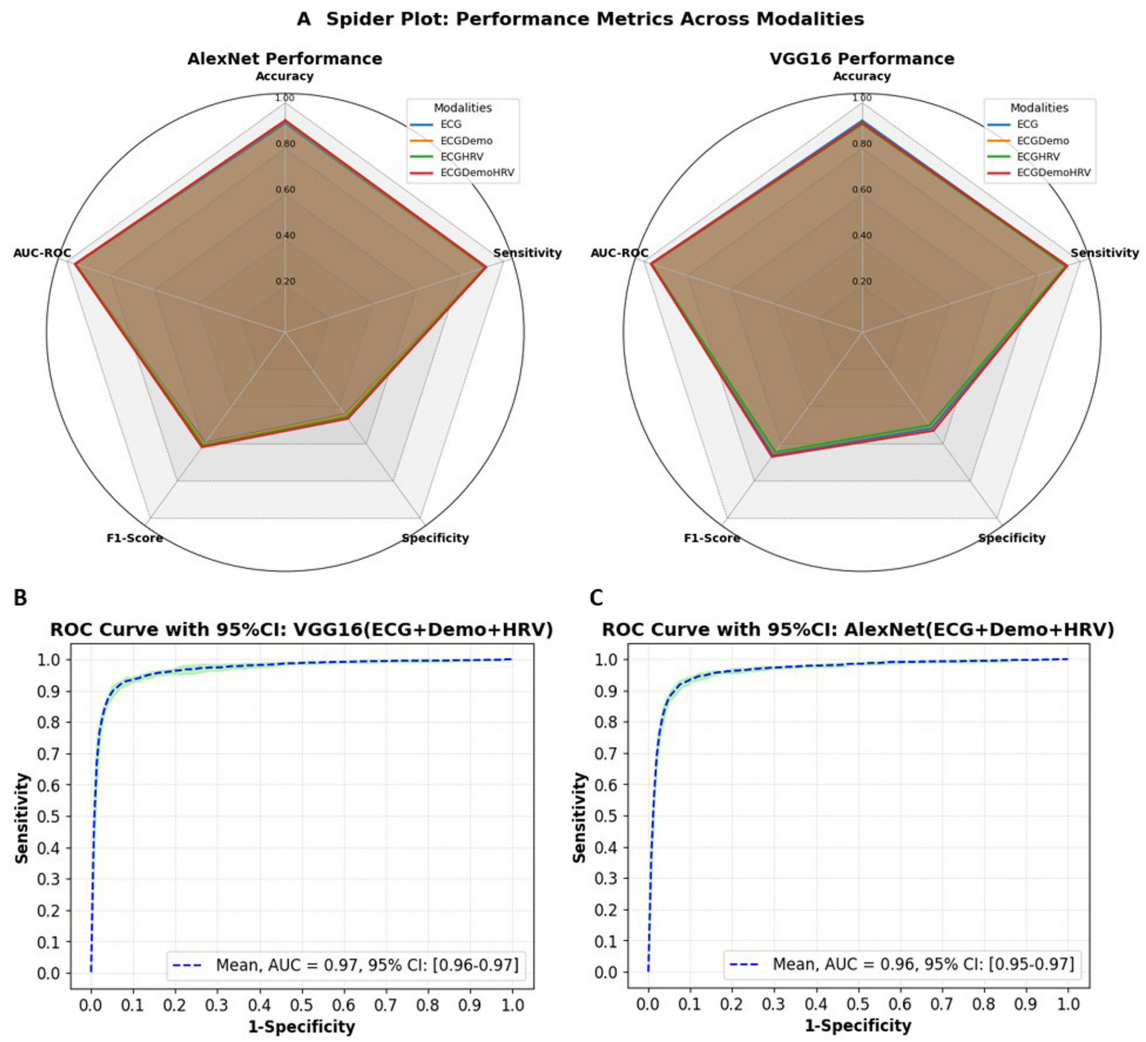
Performance comparison of AlexNet and VGG16 models across different modalities. (**A**) Spider plot showing performance metrics (accuracy, sensitivity, specificity, F1-Score and AUC-ROC) for different input combinations (ECG, ECG+Demo, ECG+HRV, and ECG+Demo+HRV). (**B**) AUC-ROC curve with 95% CI for VGG16 with the best-performing modality (ECG+Demo+HRV). (**C**) AUC-ROC curve with 95% CI for AlexNet with the best-performing modality (ECG+Demo+HRV). AUC-ROC, area under the receiver operating characteristic curve; HRV, heart rate variability.

The predictive impact of integrating CNN models with demographic information (age and sex) and HRV data was evaluated for the top-performing CNN models (with class-weight): AlexNet and VGG-16. Figure 3 provides an overview of these results (Improvements trend) across key performance metrics, including sensitivity, specificity, precision, NPV, F-Score and AUC-ROC. Actual values can be found in figure 4.

Figure 3A illustrates the improvement trends when ECG is integrated with HRV and demographic data through a multimodal approach. For AlexNet, integrating both HRV and demographic data (ECG+Demo+HRV) resulted in improvements across multiple metrics: sensitivity (from 91.18% to 92.25%), precision (from 44.26% to 46.39%), F-Score (from 0.5957 to 0.6173), specificity (from 91.41% to 92.04%) and a slight increase in AUC-ROC (from 0.9617 to 0.9629). These improvements demonstrate the positive effect of integrating HRV and demographic data with ECG, especially in enhancing sensitivity, precision, specificity and the overall F-Score. When comparing the individual contributions of ECG+Demo and ECG+HRV, ECG+HRV+Demo provided more stable improvements in performance. Adding HRV alone (ECG+HRV) increased Sensitivity and AUC-ROC slightly, while ECG+Demo improved F-Score marginally. For VGG-16, integrating HRV and demographic data (ECG+Demo+HRV) led to notable improvements in specificity (from 93.47% to 93.92%), precision (from 0.5160 to 0.5285) and F-Score (from 0.6607 to 0.6688). Additionally, AUC-ROC showed a minor increase (from 0.9669 to 0.9671). Although there was a slight decrease in sensitivity (from 92.17% to 91.13%), the overall positive changes in specificity, F-Score and AUC-ROC indicate that adding HRV and demographic data enhances the model’s ability to distinguish between AF and non-AF events, especially in the ability to correctly classify non-AF events. The detailed improvements in each of the performance metrics for both AlexNet and VGG-16, including sensitivity, specificity, precision, F-Score and AUC-ROC, are provided in the online supplemental materials under the section ‘Detailed Performance Metrics and Improvements’.

Figure 3B shows similar trends for XGBoost models, where CNN-extracted features were used for evaluation. For VGG-16, integrating HRV data (CNN_F+HRV) led to substantial improvements across multiple metrics. Sensitivity increased from 81.77% to 84.62%, while F-Score improved from 0.7466 to 0.8249, indicating better overall classification performance. Additionally, AUC-ROC rose significantly from 0.9608 to 0.9719, showing a marked improvement in model discrimination. When combining HRV and demographic data (CNN_F+Demo+HRV), the F-Score further improved (from 0.8249 to 0.8284), and precision remained stable at 0.8059, but with a slight reduction in specificity (from 0.9732 to 0.9846). These results demonstrate the positive impact of HRV and demographic data on enhancing sensitivity, F-Score and AUC-ROC, contributing to better detection of AF events. For AlexNet, the addition of HRV data (CNNF_+HRV) led to a significant increase in F-Score (from 0.7060 to 0.8019) and a noticeable improvement in AUC-ROC (from 0.9569 to 0.9715), highlighting the benefit of HRV in boosting classification accuracy. Sensitivity also improved from 75.46% to 82.10% with HRV integration, while precision increased from 0.6635 to 0.7839. Adding demographic data (CNNF_+Demo+HRV) brought further improvements, with F-Score reaching 0.8028 and AUC-ROC achieving 0.9716, indicating enhanced model performance in distinguishing both AF and non-AF events. The results show that the integration of HRV and demographic data significantly improves sensitivity, F-Score and AUC-ROC, with precision slightly improving or remaining stable.

Figure 4 summarises the performance comparison between AlexNet and VGG16 models, providing a more detailed visualisation of how HRV and demographic data (Demo) enhance the ECG signal. The spider plot in (**A**) highlights the key performance metrics, showing the variations across different input combinations (ECG, ECG+Demo, ECG+HRV and ECG+Demo+HRV). The AUC-ROC curves in (**B**) and (**C**) reflect the performance of both models when incorporating the ECG+Demo+HRV data. For AlexNet, the AUC-ROC value is 0.96, with a 95% CI ranging from 0.95 to 0.97, while VGG16 achieves an AUC-ROC of 0.97, with a 95% CI between 0.96 and 0.97. The CIs in (**B**) and (**C**) provide a clearer understanding of the robustness and reliability of the results. This figure serves as a comprehensive complement to figure 3, reinforcing the role of multimodal input in improving model performance and demonstrating the discriminative power of these models with additional data.

Online supplemental figure 2 provides a comprehensive overview of the performance impact across different datasets (PLBXL, CPSC and Georgia) when integrating ECG, HRV and demographic data. As seen in the figure, the impact of data integration varies notably between the datasets, with PLBXL showing the most consistent improvements across most of the metrics, particularly in VGG16. In contrast, CPSC showed only modest gains in Sensitivity and F-Score, suggesting that the added data had a lesser effect on this dataset. Meanwhile, for the Georgia dataset, the combined integration of ECG, HRV and demographic data led to consistent improvements across metrics, particularly in F-Score and sensitivity, similar to PLBXL, though the improvements were more subtle. These differences underline that the benefits of integrating HRV and demographic data are influenced by the dataset’s characteristics.

### Saliency map

**Figure 5:**
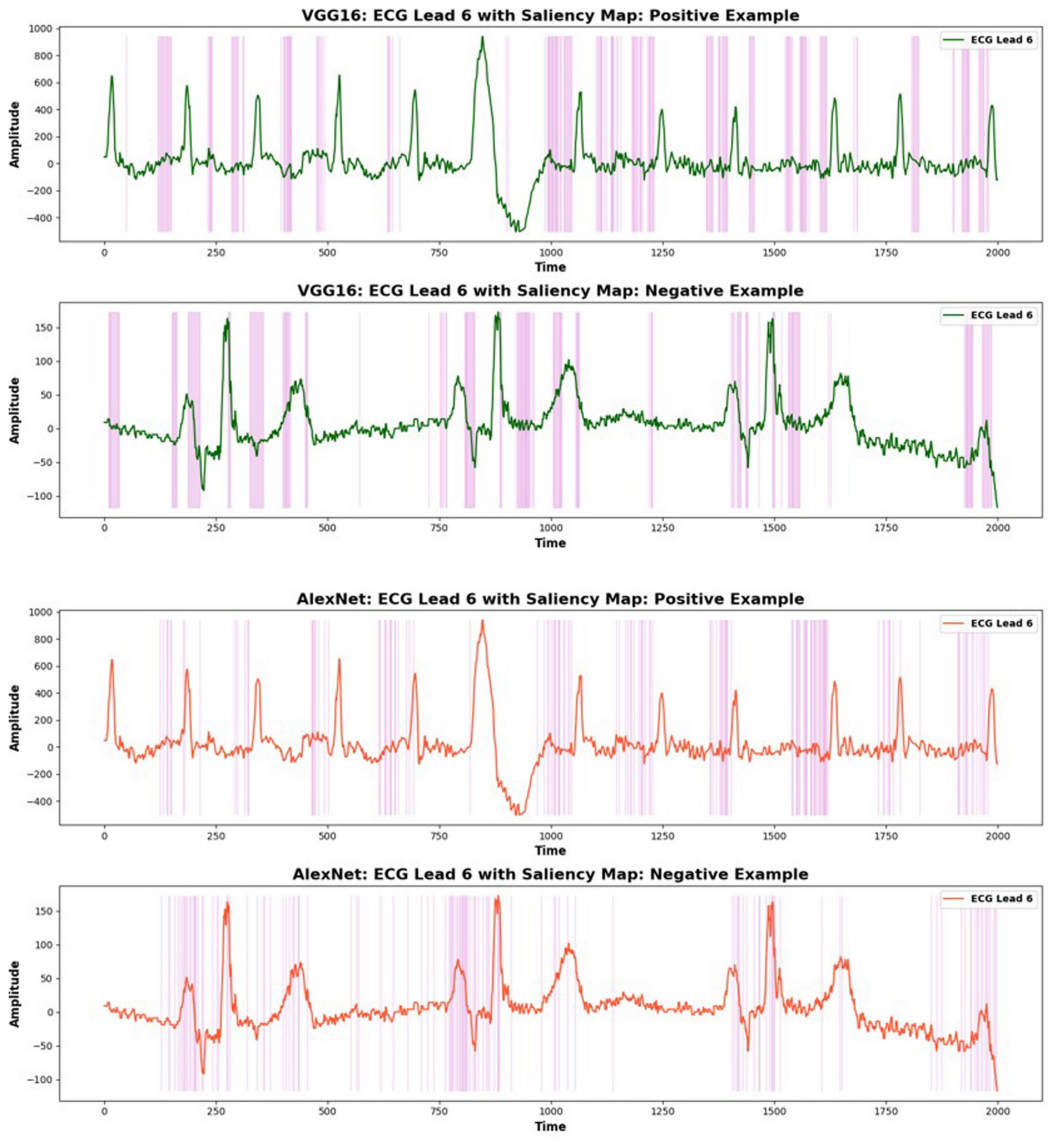
Saliency maps for both positive and negative samples from VGG16 and AlexNet for lead 6. The maps highlight the model’s focus on key areas, such as the absence of the P-wave, to aid in decision-making.

In figure 5, saliency maps generated by VGG16 and AlexNet are shown for both positive (AF) and negative (non-AF) samples. These maps highlight areas of the ECG signal that the models focused on for detecting AF. For positive samples, the models focused on the absence of the P-wave, a critical feature for identifying AF. In contrast, for negative samples, the models emphasised regular sinus rhythm patterns, including well-defined P-waves.

The saliency map is shown on lead V6, which was selected for its clear view of the left atrium’s electrical activity. This lead reliably represents atrial and left ventricular abnormalities, making it essential for AF detection. Both VGG16 and AlexNet demonstrated their ability to focus on key features like the P-wave and atrial activity, reinforcing their effectiveness in AF detection.

## Discussion

This study explored the integration of ECG signals with HRV and demographic data (age and sex) to predict AF. By combining these data sources, we aimed to unlock the full potential of predictive models. The results confirm that multimodal approaches provide substantial improvements across several performance metrics. Additionally, we investigated whether the final prediction could be enhanced by stacking another ML model, extreme gradient boosting (XGBoost), on top of the features extracted by the neural network. These findings demonstrate that it is possible to create a powerful ML model for predicting AF, even with relatively small training datasets. The results also emphasise the importance of adopting a broad, integrative strategy when developing predictive models. The overall best performing models were the AlexNet and VGG-16, with multimodal input including both age, sex and HRV parameters. This was not demonstrated earlier.

The best-performing models were simpler architectures like AlexNet and VGG-16, contrary to expectations of superior performance from more complex models like Transformers or ResNet. This discrepancy may stem from the larger data requirements of complex architectures to optimise performance. Transformers, for instance, include attention mechanisms well-suited for ECG interpretation but require extensive data due to their higher parameter count. Similarly, ResNet and inception may have underperformed due to the trade-off between model complexity and the available dataset size of around 35 000 cases. This highlights the effectiveness of simpler models when data is limited.

Both AlexNet and VGG-16 exhibited strong performance in the ECG-only models, but the inclusion of HRV and demographic data markedly enhanced their predictive capabilities. AlexNet, with ECG data alone, achieved a sensitivity of 91.17%, specificity of 91.41%, precision of 44.26% and an AUC-ROC of 0.9617. However, when HRV and demographic data (ECG+Demo+HRV) were integrated, substantial improvements were observed: sensitivity increased to 92.25%, precision rose to 46.39% and AUC-ROC improved slightly to 0.9629. The overall F-Score also increased to 0.6173, while specificity remained high at 92.03%. Similarly, VGG-16 demonstrated notable improvements when HRV and demographic data were added. Specificity improved from 93.47% to 93.92%, and the F-Score rose from 0.6607 to 0.6688, while the AUC-ROC saw a minor increase from 0.9669 to 0.9671. Although there were slight decreases in sensitivity and precision, the overall improvements in specificity and F-Score highlight the robustness of multimodal integration. The overall robustness of multimodal integration highlights the benefit of incorporating multiple data streams to boost predictive performance. Sensitivity remains a top priority in medical settings, where it is critical to minimise false negatives, which can have severe clinical implications. Thus, emphasising sensitivity in these models ensures a balance between precision and recall. These findings emphasise the effectiveness of integrating additional data streams to enhance predictive performance.

The superior performance of AlexNet and VGG-16 could also be attributed to their robustness against class imbalance and their ability to maintain performance across various metrics without requiring class weighting. In medical settings, where reducing false negatives is critical, these models effectively balanced sensitivity with PPV. The inclusion of HRV and demographic data (ECG+Demo+HRV) further solidified their advantage. Saliency map visualisations reinforced the reliability of these models by highlighting clinically relevant features, such as the absence of the P-wave, in decision-making. While some studies^44^ suggest that Grad-CAM may focus on irrelevant ECG regions when models are pretrained on non-ECG tasks, our models were specifically fine-tuned for ECG classification, likely mitigating such issues. However, saliency maps alone should not be considered definitive proof of model efficacy and must be evaluated alongside traditional performance metrics.

Discrepancies in model performance across datasets (PLBXL, CPSC, Georgia) further underscore the importance of high-quality, well-labelled datasets to ensure consistent generalisability.

The efficacy of multimodal integration was corroborated using XGBoost models. Combining HRV and demographic data (CNN_F+Demo+HRV) consistently enhanced performance metrics across architectures. For VGG-16, this integration improved the F-Score from 0.7465 to 0.8284, while precision rose from 0.6871 to 0.8059, and AUC-ROC increased from 0.9608 to 0.9721. Similarly, for AlexNet, combining HRV and demographic data (CNNF_+Demo+HRV) boosted the F-Score to 0.8028 and improved the AUC-ROC to 0.9716, with sensitivity rising to 82.10% and precision to 0.7849. These results underscore the synergistic value of incorporating HRV and demographic data with ECG, yielding robust gains in classification accuracy and sensitivity.

We did not expect the HRV parameters to improve the model’s performance significantly. Neural networks are remarkably capable of capturing non-linear functions and subtle signals in the data, which should include HRV parameters. Adding HRV data improved both the baseline CNN model and the model fused with extreme gradient boosting. This suggests that although neural networks are very capable of learning complex patterns from raw data, the way data are represented can significantly impact their performance and the patterns the network deciphers. By including HRV metrics, we are effectively providing the network with additional features that may represent additional underlying patterns which the model might not easily learn from the raw ECG signal alone. We believe future efforts to develop DL models for AF should include this multimodal input in order to improve the model performance.

Hence, we are likely to observe a human assessment of the vast majority of AI assessments in the coming decades, until the underlying evidence suggests that AI has surpassed human capability. Second, it can be difficult to decisively determine whether a rhythm is abnormal using a 10 s recording. The arrhythmia may be brief, and other disturbances (including other arrhythmias, extrasystoles, noise, etc) may render the interpretation very difficult, even for the most experienced electrophysiologist. Hence, researchers and clinicians should keep in mind that short ECG recordings may never offer the fully automated interpretation promised by AI. This should be recognised by manufacturers, since these machines could easily use a longer ECG recording as the standard setting. Third, physician offices could not easily accommodate the additional work of checking several false positives.

With regard to multimodal neural networks, we believe that this should be the standard approach to all neural networks.^45^ Probabilities in medicine are rarely unimodal, and researchers should strive for using multimodal data to improve the final performance.

When contextualising our work in relation to existing studies^25^,^27^ focusing on 12-Lead ECG analysis, our study evaluates publicly available datasets, considering variability in data sources and exploring various scenarios. We observed that while using a single data source, such as PTB-XL, yielded excellent results across all metrics, performance decreased when combining multiple data sources. This highlights the challenge of dealing with highly unbalanced data, where complex models may struggle to perform well for rare classes despite achieving high overall accuracy. Most existing studies have emphasised complex models, neglecting simpler yet efficient alternatives in terms of implementation and time efficiency. Additionally, we investigated how incorporating additional information like HRV can further enhance performance across evaluation metrics.

Baek *et al*^25^ analysed a total of 2412, 12-lead ECGs, demonstrated performance on internal and external datasets from Inha University Hospital, achieving an AUC-ROC curve of 0.79 and 0.75, sensitivity of 82% and 77%, specificity of 78% and 72%, and overall accuracy of 72.8% and 71.2%, respectively. Cal *et al*^26^ proposed a deep densely connected neural network for AF detection in ECG waveforms, using 16 557 recordings from multiple hospitals and wearable ECG devices, achieving an accuracy of 99.35%±0.26%, sensitivity of 99.19%±0.31% and specificity of 99.44%±0.17%.Couceiro *et al*^27^ extracted features including heart rate analysis and P-wave detection, using support vector classification on databases such as the St.-Petersburg Institute of Cardiological Technics 12-lead Arrhythmia Database and Cardiorisk. They reported sensitivity of 88.5% and specificity of 92.9%.

Stoyanov *et al*^46^ explored the effectiveness of transfer learning for AF detection using colormap-based Holter ECG representations. Their study applied deep transfer learning to 18 pretrained DNNs, achieving validation accuracies between 94.5% and 97.4% during retraining and further improving to 98%–98.8% with fine-tuning. The final models demonstrated high accuracy (96.3%–97.6%) and improved sensitivity (from 88.2%–93.9% to 93.4%–95.8%) after fine-tuning. Unlike their approach, which leveraged pretrained models and colormap-based ECG representations, our study directly applied CNN architectures trained from scratch as 1D CNN models on raw ECG signals.

In contrast to existing work that primarily evaluated AF detection methods on private datasets, our study used publicly available databases, encompassing a total of 35 634 (10s: 33532) recordings. This approach accounted for significant variability within the data and demonstrated excellent discrimination, achieving a sensitivity of 0.9224, along with effective delineation through saliency maps. The incorporation of HRV features into our multimodal framework significantly enhanced the accuracy and reliability of AF detection, underscoring the value of integrating physiological data into predictive models. Furthermore, the use of diverse public ECG datasets contributed to the robustness and generalisability of our findings, ensuring that the models could adapt to different patient populations.

Our results revealed that simpler models, such as AlexNet and VGG16, were not only efficient but also delivered competitive performance, providing interpretable saliency maps that enhance clinical understanding. However, there are limitations to consider. The datasets employed, though diverse, may not fully capture the complexities encountered in real-world clinical practice, and the study does not differentiate between symptomatic and asymptomatic AF cases. Additionally, the retrospective nature of the study, potential overfitting and variability in labelling quality across datasets (some labels were machine-generated, while others were reviewed or adjudicated by cardiologists) require further validation in prospective, real-world settings and on independent datasets. Moreover, data integration from multiple sources introduces possible differences in acquisition protocols that may slightly affect generalisability. Therefore, additional research is needed to assess the generalisability of these models across a wider range of patient conditions, ensuring their effectiveness in diverse clinical scenarios.

To conclude, we studied a wide array of neural network architectures and assessed the value of integrating demographic data and HRV parameters for enhancing the prediction of AF. Simpler models like AlexNet and VGG-16 outperformed more complex architectures like ResNet and Transformers. Adding HRV data improves the prediction of AF/AFL. The multimodal AlexNet could offer clinicians a very high sensitivity but requires human assessment to confirm cases of AF/AFL.

## supplementary material

10.1136/openhrt-2025-003185 online supplemental file 1

## Data Availability

Data are available in a public, open access repository.
