# Supplementary material for: Integrating deep learning with ECG, heart rate variability and demographic data for improved detection of atrial fibrillation
Source: Open Heart. 2025 Mar 31;12(1):e003185. doi: 10.1136/openhrt-2025-003185 (PMC11962809; doi:10.1136/openhrt-2025-003185)
Supplement: online supplemental file 1 [file openhrt-12-1-s001.docx]

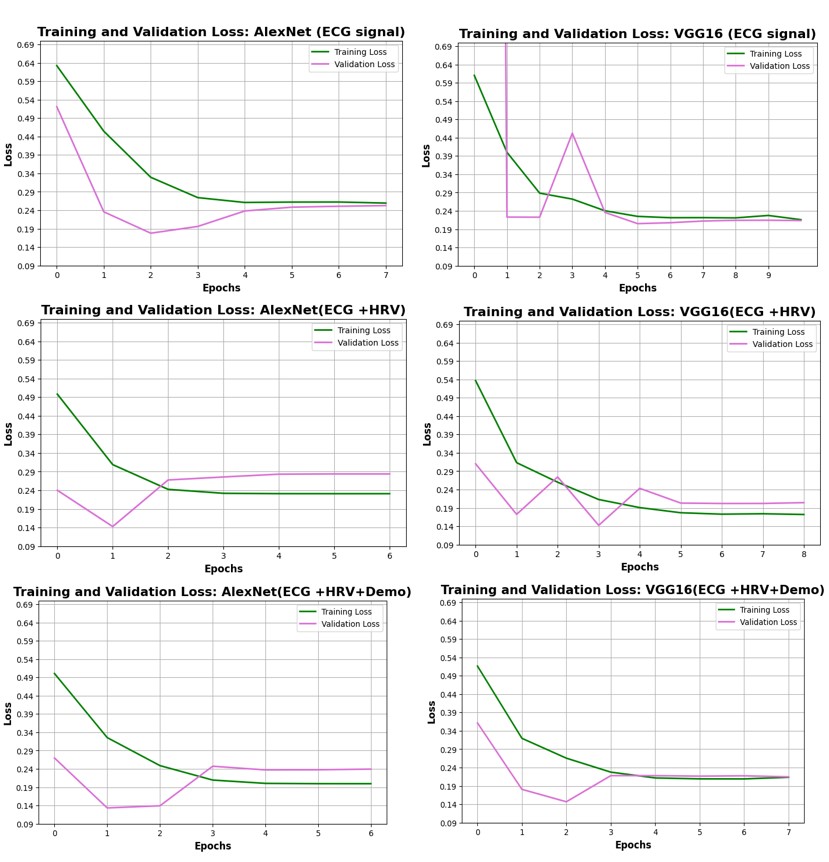


Supplementary Figure 1. Comparison of training loss progression: Average training and validation loss for AlexNet and VGG-16 across different data combinations, evaluated through five-fold cross-validation.


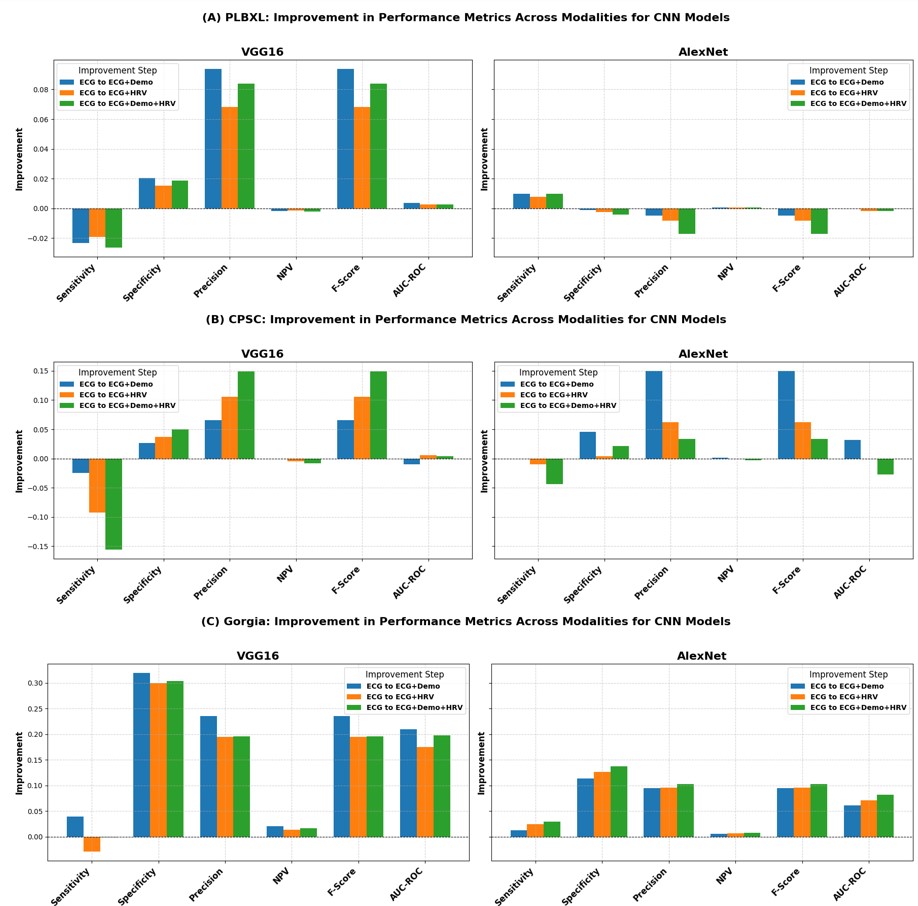


Supplementary Figure 2. Performance improvements: Impact of integrating ECG, HRV, and demographic data on model performance, across key metrics for the PTBXL, CPSC, and Georgia datasets.

- **Supplementary Table 1: Dataset Overview and Train-Validation-Test Split.**

| **Dataset Overview** | | | | | | | |
| --- | --- | --- | --- | --- | --- | --- | --- |
| **Dataset** | **Number of Recordings** | **Mean Duration (s)** | **Mean Age (years)** | **Sex (Male/**  **Female)** | **10s ECG Segments** | | **Sample Frequency (Hz)** |
|  |  |  |  |  | **AF/AFL Cases** | **Non-AF Cases** |  |
| **PTB-XL** | 21,837 | 15.9 | 63.7 | 53% / 46% | 1570 | 20267 | 500 |
| **CPSC Extra** | 3,453 | 10.0 | 59.8 | 52% / 48% | 107 | 1296 | 500 |
| **Georgia 12-Lead ECG** | 10,344 | 10.0 | 60.5 | 54% / 46% | 739 | 9553 | 500 |
| **Total** | 35,634 |  |  |  | 2416 | 31116 |  |
|  | **Train-Validation-Test Split of Total 10s ECG Segments Used (33,532)** | | | | | | |
| **Training (80%)** | 26825 | - | - | - | \| 1,932 \| \| --- \| | \|  \| \| --- \|   24,893 | - |
| **Validation (20% of Training)** | 6609 | - | - | - | \| 386 \| \| --- \| | \|  \| \| --- \|   6,223 | - |
| **Testing (20%)** | 5463 | - | - | - | \| 484 \| \| --- \| | \|  \| \| --- \|   4,979 | - |

*This table summarizes the key characteristics of the datasets used in this study, including the number of recordings, mean duration, mean age, sex distribution, the number of 10-second ECG segments used, and the sample frequency (500 Hz). Additionally, it details the train-validation-test split of the total 10-second ECG segments, maintaining the stratified proportions of AF/AFL and Non-AF cases for training, validation, and testing phases.*

- **Details Related to HRV Feature Calculation**

1. **Filtration**

Each ECG lead was individually processed to compute heart rate variability (HRV) features. To ensure robustness, we selected the lead with the median heart rate across 12 leads, avoiding unreliable estimates caused by noise or low amplitude. To ensure reliable HRV calculations, we applied the following inclusion criteria:

- The computed median heart rate must not be NaN.
- The median heart rate must be within a physiologically plausible range (30–300 bpm).

1. **HRV Parameters & Equations [*Ref 1*]**

The heart rate for each lead was computed using **HeartPy [*Ref 2*]**, and the selected HRV parameters were integrated into the multimodal models to assess their added contribution beyond neural network-extracted features. The HRV parameters extracted include the following, and here are the equations [***Ref 3***] for them:

1. **Heart Rate (HR):** **The heart rate is the number of heartbeats per minute.**

$$Heart Rate=\frac{60}{Average RR interval (in seconds)}$$

Where the RR interval is the time between two successive R-peaks in the ECG signal.

1. **Inter-Beat Interval (IBI):** **The inter-beat interval (IBI) is the time between two successive heartbeats (R-R intervals).**

$${IBI}_{i}=t_{R\left( i \right)}-t_{R(i-1)}$$

Where $t_{R(i)}$is the time of the *i-th* R-peak, and $t_{R(i-1)}$ is the time of the previous R-peak.

1. **SDNN (Standard Deviation of NN intervals): The SDNN is the standard deviation of the normal-to-normal (NN) intervals, i.e., the time between successive R-wave peaks.**

$$\boldsymbol{SDNN=}\sqrt{\frac{\boldsymbol{1}}{\boldsymbol{N-1}}\sum_{\boldsymbol{i=1}}^{\boldsymbol{N}} \left( \boldsymbol{N}\boldsymbol{N}_{\boldsymbol{i}}\boldsymbol{-}\bar{\boldsymbol{NN}} \right)^{\boldsymbol{2}}}$$

Where ${NN}_{i}$is each individual RR interval, $\bar{NN}$ is the mean RR interval, and N is the total number of RR intervals.

1. **RMSSD (Root Mean Square of Successive Differences): RMSSD is the square root of the mean of the squares of the successive differences between adjacent RR intervals.**

$$\boldsymbol{RMSDD=}\sqrt{\frac{\boldsymbol{1}}{\boldsymbol{N-1}}\sum_{\boldsymbol{i=2}}^{\boldsymbol{N}} \left( \boldsymbol{N}\boldsymbol{N}_{\boldsymbol{i}}\boldsymbol{-N}\boldsymbol{N}_{\boldsymbol{i-1}} \right)^{\boldsymbol{2}}}$$

Where ${NN}_{i}$and ${NN}_{i-1}$are successive RR intervals.

1. **PNN20 (Percentage of NN Intervals Differing by More Than 20 ms): PNN20 is the percentage of successive RR intervals that differ by more than 20 milliseconds.**

$$PNN20=\frac{Number of successive RR intervals where \mid{NN}_{i}-{NN}_{i-1}\mid>20 ms}{N-1}*100$$

Where ${NN}_{i}$and ${NN}_{i-1}$are successive RR intervals.

6**.** **PNN50 (Percentage of NN Intervals Differing by More Than 50 ms): PNN50 is the percentage of successive RR intervals that differ by more than 50 milliseconds.**

$$PNN50=\frac{Number of successive RR intervals where \mid{NN}_{i}-{NN}_{i-1}\mid>50 ms}{N-1}*100$$

Where ${NN}_{i}$and ${NN}_{i-1}$are successive RR intervals.

1. **HR MAD (Heart Rate Mean Absolute Deviation): HR MAD is the mean absolute deviation of the heart rate from its mean value.**

$$HR MAD=\frac{1}{N} \sum_{i=1}^{N} \left| HR_{i}- \bar{HR} \right|$$

Where ${HR}_{i}$is the heart rate at the *i-th* instance, and $\bar{HR}$ is the mean heart rate over the duration of the measurement.

***Ref 1: Shaffer, Fred, and Jay P. Ginsberg. "An overview of heart rate variability metrics and norms." Frontiers in public health 5 (2017): 258.***

***Ref 2: Van Gent, Paul, et al. "HeartPy: A novel heart rate algorithm for the analysis of noisy signals." Transportation research part F: traffic psychology and behaviour 66 (2019): 368-378.***

***Ref 3: Moses, Jeban Chandir, et al. "Time‐domain heart rate variability features for automatic congestive heart failure prediction." ESC Heart Failure 11.1 (2024): 378-389.***

- **Performance Metrics and Model Interpretability**

Model Performance Metrics

1. Sensitivity (Recall or True Positive Rate - TPR)
   - Measures the ability of the model to correctly identify positive cases.
   - Equation: Sensitivity=TP/(TP+FN)
   - Interpretation: Higher sensitivity indicates fewer false negatives, meaning the model effectively detects actual positive cases.
2. Specificity (True Negative Rate - TNR)
   - Measures how well the model identifies negative cases.
   - Equation: Specificity=TN/(TN+FP)
   - Interpretation: A high specificity means fewer false positives, ensuring the model does not misclassify negatives as positives.
3. Positive Predictive Value (PPV or Precision)
   - Measures the proportion of predicted positives that are actually correct.
   - Equation: PPV=TP/(TP+FP)
   - Interpretation: A higher PPV means the model’s positive predictions are more reliable.
4. Negative Predictive Value (NPV)
   - Measures the proportion of predicted negatives that are actually correct.
   - Equation: NPV=TN/(TN+FN)
   - Interpretation: A higher NPV means the model is more accurate in predicting negative cases.
5. F1-Score
   - The harmonic mean of precision (PPV) and recall (sensitivity). It balances both metrics.
   - Equation: F1=2×(PPV×Sensitivity)/(PPV+Sensitivity)
   - Interpretation: A higher F1-score indicates a good balance between precision and recall.
6. Area Under the ROC Curve (AUC-ROC)
   - Measures the model’s ability to distinguish between classes across different classification thresholds. Interpretation: A higher AUC (closer t1) means the model performs well in separating positive and negative cases.

7. Accuracy:

Accuracy =(TP+TN)/(TP+TN+FP+FN)

**where:**

- **TP = True Positives, TN = True Negatives**
- **FP= False Positives, FN = False Negatives**
- **Detailed Performance Metrics and Improvements**

The following tables summarize the detailed performance metrics for **AlexNet** and **VGG-16**, including Sensitivity, Specificity, Precision, Negative Predictive Value (NPV), Positive Predictive Value (PPV), False Negative Rate, F-Score, and AUC-ROC. The improvements across different input combinations (ECG, ECG+Demo, ECG+HRV, and ECG+Demo+HRV) are also highlighted.

**Table S1: AlexNet Performance Metrics**

| **Model** | **Sensitivity (%)** | **Specificity (%)** | **Precision (%)** | **Negative Predictive Value (%)** | **Positive Predictive Value (%)** | **False Negative Rate (%)** | **F-Score** | **AUC-ROC** |
| --- | --- | --- | --- | --- | --- | --- | --- | --- |
| **ECG** | 91.18 | 91.41 | 44.26 | 99.28 | 44.26 | 8.82 | 0.5957 | 0.9617 |
| **ECG + Demo** | 91.92 | 91.43 | 44.62 | 99.34 | 44.62 | 8.08 | 0.6003 | 0.9619 |
| **ECG + HRV** | 92.16 | 91.70 | 45.44 | 99.37 | 45.44 | 7.83 | 0.6084 | 0.9623 |
| **ECG + Demo + HRV** | 92.25 | 92.04 | 46.39 | 99.38 | 46.39 | 7.75 | 0.6173 | 0.9629 |

**Table S2: VGG-16 Performance Metrics**

| **Model** | **Sensitivity (%)** | **Specificity (%)** | **Precision (%)** | **Negative Predictive Value (%)** | **Positive Predictive Value (%)** | **False Negative Rate (%)** | **F-Score** | **AUC-ROC** |
| --- | --- | --- | --- | --- | --- | --- | --- | --- |
| **ECG** | 92.17 | 93.47 | 51.60 | 99.37 | 51.60 | 7.83 | 0.6607 | 0.9669 |
| **ECG + Demo** | 90.96 | 93.27 | 50.29 | 99.28 | 50.29 | 9.03 | 0.6475 | 0.9659 |
| **ECG + HRV** | 90.76 | 93.19 | 50.01 | 99.27 | 50.01 | 9.23 | 0.6444 | 0.9663 |
| **ECG + Demo + HRV** | 91.13 | 93.92 | 52.85 | 99.30 | 52.85 | 8.87 | 0.6688 | 0.9671 |

These metrics demonstrate the incremental improvements in model performance when integrating HRV and demographic data (age and sex) into the ECG signal.
